# Tongue nodules in canine leishmaniosis — a case report

**DOI:** 10.1186/1756-3305-5-120

**Published:** 2012-06-15

**Authors:** Carlos Viegas, João Requicha, Carlos Albuquerque, Teresa Sargo, João Machado, Isabel Dias, Maria A Pires, Lenea Campino, Luís Cardoso

**Affiliations:** 1 Department of Veterinary Sciences, School of Agrarian and Veterinary Sciences, University of Trás-os-Montes e Alto Douro (UTAD), Vila Real, Portugal; 2 Department of Polymer Engineering, 3B’s Research Group – Biomaterials, Biodegradables and Biomimetics, University of Minho, Guimarães, Portugal; 3 Centre of Genetics and Biotechnology – Institute for Biotechnology and Bioengineering, UTAD, Vila Real, Portugal; 4 Veterinary Hospital, UTAD, Vila Real, Portugal; 5 CECAV – Veterinary and Animal Science Centre, UTAD, Vila Real, Portugal; 6 Leishmanioses Laboratory, Medical Parasitology RTU, Institute of Hygiene and Tropical Medicine, Lisbon, Portugal; 7 Parasite Disease Group, IBMC – Instituto de Biologia Celular e Molecular, University of Oporto, Oporto, Portugal

**Keywords:** Canine leishmaniosis, Dog, Glossitis, Nodules, Oral cavity, Portugal, Tongue

## Abstract

**Background:**

Canine leishmaniosis (CanL) caused by *Leishmania infantum* is an endemic zoonosis in southern European countries. Infected dogs can present rare or atypical forms of the disease and diagnosis can be challenging. The present report describes a case of tongue nodules in a 3-year-old neutered female Labrador Retriever dog with leishmaniosis.

**Findings:**

A fine needle aspiration of the lingual nodules revealed amastigote forms of *Leishmania* inside macrophages. Differential diagnosis ruled out neoplasia, *calcinosis circumscripta*, solar glossitis, vasculitis, amyloidosis, eosinophilic granulomas, chemical and electrical burns, uremic glossitis and autoimmune diseases. Combined therapy with antimoniate meglumine and allopurinol for 30 days resulted in the normalization of hematological and biochemical parameters. Two months after diagnosis and the beginning of treatment, a mild inflammatory infiltrate was observed by histopathology, but an anti-*Leishmania* immunofluorescence antibody test (IFAT) was negative as well as a PCR on both tongue lesions and a bone marrow aspirate. Seven months after diagnosis, the dog’s general condition appeared good, there were no tongue lesions and a new IFAT was negative. Fifteen months after diagnosis this clinically favourable outcome continued.

**Conclusions:**

The dog could have suffered a relapsing episode of CanL, but a new systemic or local infection cannot be excluded. Regular clinical re-evaluation should be maintained, as a future relapse can potentially occur. In conclusion, CanL should be considered in the differential diagnosis of nodular glossitis in dogs.

## Findings

Canine leishmaniosis (CanL) caused by *Leishmania infantum* is a zoonotic parasitic disease endemic in southern European countries [1,2]. The pathogeny of CanL is mainly due to pseudogranulomatous inflammation and deposition of immune complexes in cutaneous and visceral tissues, with clinical presentations of chronic and immunosuppressive disease [3]. Dogs infected with *L. infantum* can present rare or atypical forms of leishmaniosis [4]. These include a few reported cases of single [5,6] or multiple tongue nodules [7,8] and ulcers of the lingual mucosa [4,9].

**Figure 1:**
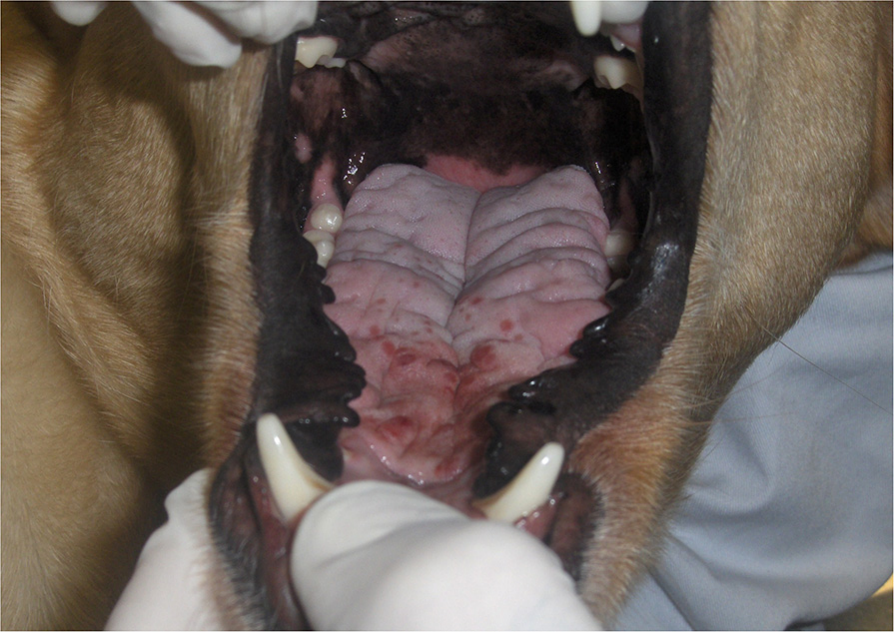
**Dorsal surface of the tongue before the beginning of treatment.** Multiple and coalescent reddish nodular lesions were seen on the rostral portion.

This report describes a case of tongue nodules in a 3-year-old neutered female Labrador Retriever dog with a diagnosis of leishmaniosis. Diagnosis was carried out two years previously and was based on a positive (titre of 1:80; cut-off titre of 1:80) immunofluorescence antibody test (IFAT) to *Leishmania* and detection of amastigotes in bone marrow. The dog was treated with meglumine antimoniate (75 mg/kg subcutaneously once a day) plus allopurinol (10 mg/kg orally twice a day) for 30 days and with allopurinol alone (same dosage) for six subsequent months. A regression of clinical signs (ocular signs, disseminated hair loss and dry seborrhoea) and of clinico-pathological alterations (increased blood urea nitrogen [52 mg/dl; reference range: 7–32 mg/dl], increased creatinine [1.99 mg/dl; reference range: 0.5-1.4 mg/dl] and increased urine protein:creatinine [UP:C] ratio [2.4; reference range < 0.2]) was attained after this course of treatment. In a follow-up two years after the initial diagnosis of CanL the dog was in good body condition, alert, hydrated, afebrile, with only mild hair loss and mild dry seborrhea, and without other systemic signs. The owner mentioned the dog was having difficulties with food prehension and chewing. Physical examination of the oral cavity revealed halitosis, generalized gingivitis, ulcerative glossitis and some reddish, soft nodules with 2–9 mm in diameter on the lingual dorsal surface (Figure 1). Differential diagnosis with other nodular and ulcerative diseases of tongue included neoplastic processes [10], *calcinosis circumscripta*[11], solar glossitis [12], vasculitis [13], amyloidosis [14], eosinophilic granuloma [15], chemical and electrical cord burns, uremic glossitis [14] and autoimmune diseases (systemic lupus erythematosus, *pemphigus vulgaris*) [13,14]. A relapse of leishmaniosis with a new clinical presentation was also considered due to the previous diagnosis.

IFAT for antibodies to *Leishmania* provided a negative result (titre of 1:40; cut-off titre of 1:80). Complete blood count revealed severe leukopenia (1.4 × 10^9^/L; reference range: 6-17 × 10^9^/L) associated to severe neutropenia (0.68 × 10^9^/L; reference range: 3–11.5 × 10^9^/L) and mild thrombocytopenia (182 × 10^9^/L; reference range: 250-500 × 10^9^/L); and serum biochemical analysis moderate alanine aminotransferase increase (157 IU/L; reference range: 0–130 IU/L). A mild proteinuria was found (10 mg/L) for a urine creatinine level of 1076 mg/L (UP:C ratio: 0.009; reference range < 0.2). Microscopic examination revealed a normal or inactive urine sediment. Serum protein levels were normal (albumin: 2.8 g/dl; reference range: 2.8-3.02 g/dl; globulins: 3.4 g/dl; reference range: 2.8-3.6 g/dl). Measurement of antinuclear antibodies (ANA) gave a titre below 1:40, which is regarded as normal.

**Figure 2:**
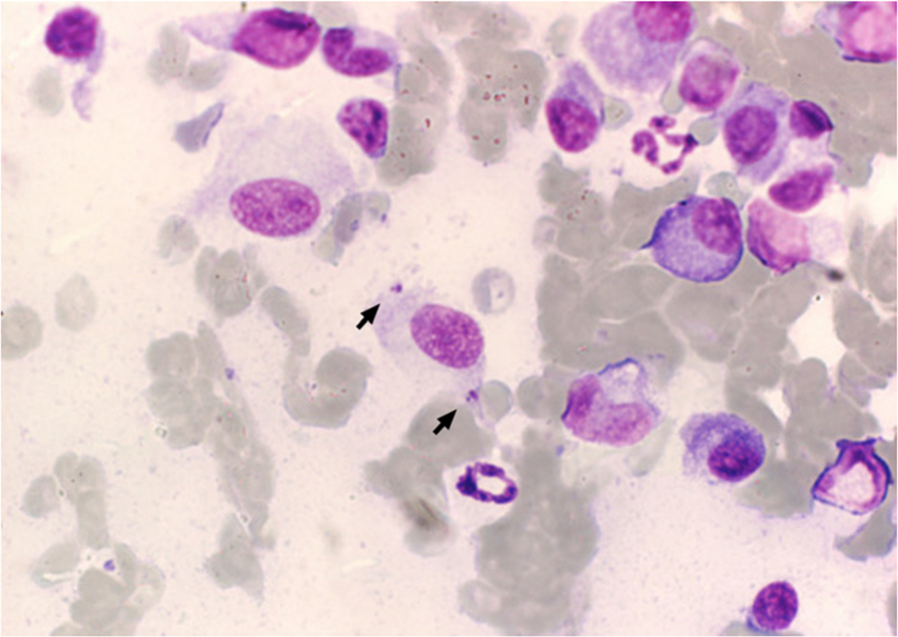
**Cytological sample from one lingual nodule (**Figure1 **).** Arrows: two intra-macrophagic *Leishmania* amastigotes (Giemsa; 1000×).

The tongue lesions appeared unchanged after seven days of a large spectrum antibiotic treatment with spiramycin plus metronidazole (75,000 IU/kg and 12.5 mg/kg orally once a day, respectively). A fine needle aspiration of the tongue nodules was done under appropriate sedation and microscopic observation of stained smears revealed amastigotes of *Leishmania* spp. inside macrophages (Figure 2). Uremic glossitis was discarded by serum biochemistry; and autoimmune diseases by negative specific serology (ANA).

**Figure 3:**
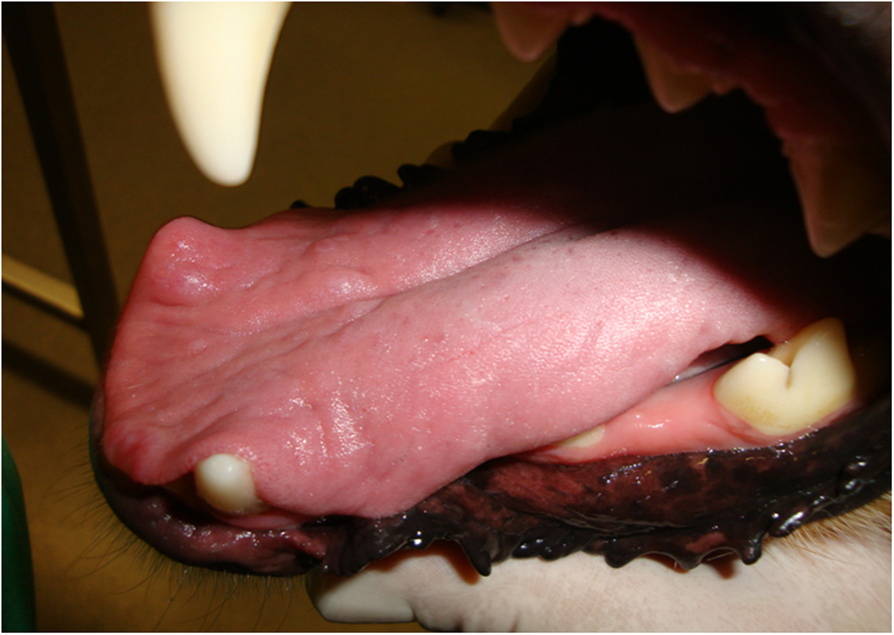
**Dorsal surface of the tongue at day 10.** A few reddish flattened lesions were seen after 10 days of combined treatment with meglumine antimoniate and allopurinol.

**Figure 4:**
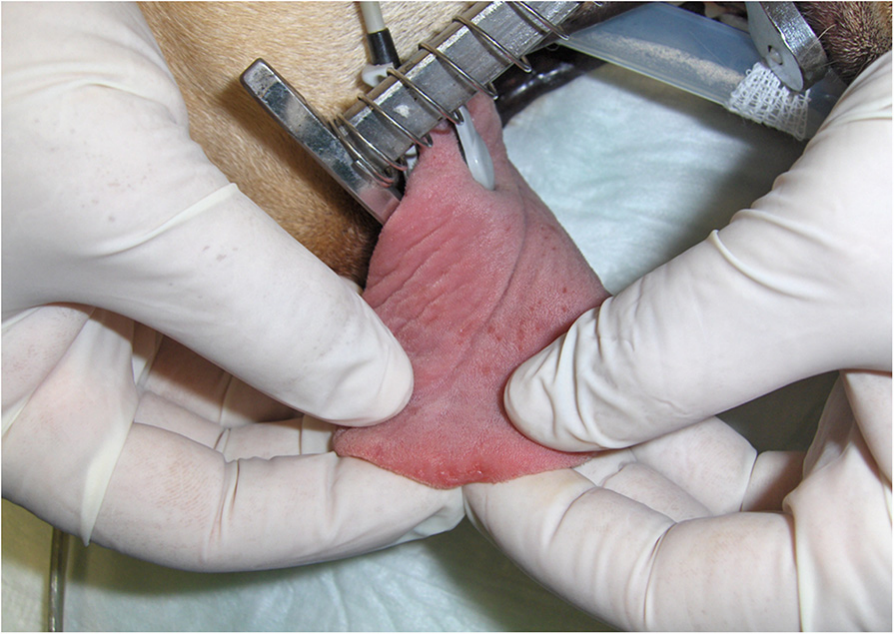
**Dorsal surface of the tongue at day 60.** Some remnant erosive lesions on the edge were seen one month after the end of combined treatment. Two of these lesions were sampled for histopathology (Figure 5) and PCR.

**Figure 5:**
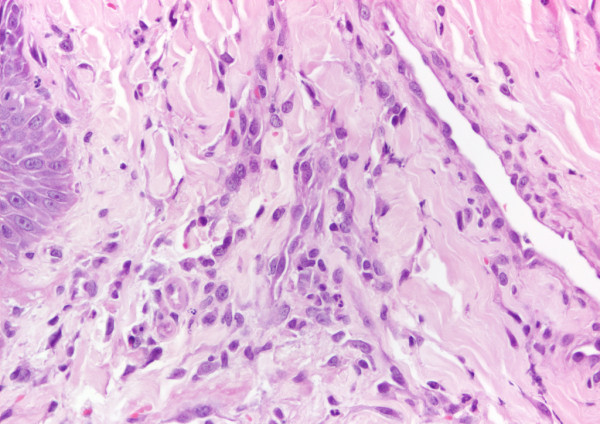
**Histopathological image of an erosive lesion (**Figure 4 **).** A mild inflammatory infiltrate mainly comprising plasma cells, macrophages, a few eosinophils and neutrophils (H&E; 400×) was observed one month after the end of combined treatment.

**Figure 6:**
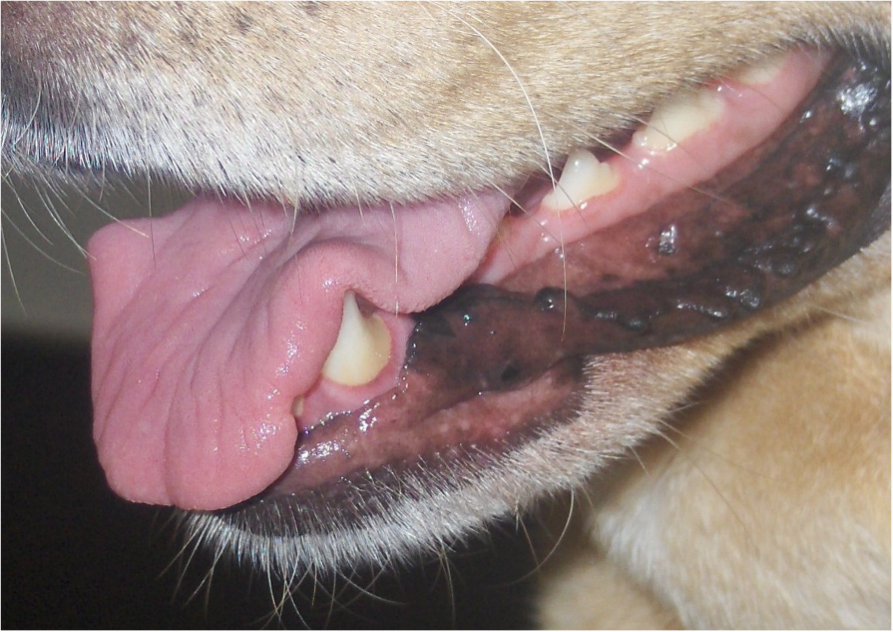
**Dorsal surface of the tongue at day 210.** Complete healing of macroscopic lesions at the end of treatment with allopurinol alone (seven months after diagnosis).

The dog was given another course of meglumine antimoniate and allopurinol for 30 days and allopurinol alone for an additional six-month period. Ten days after diagnosis and the start of combined treatment the lesions apparently improved (Figure 3) and the dog had normal blood and urine analyses. At completion of the meglumine antimoniate and allopurinol combined 30-day course, macroscopic regression of the tongue nodules was observed, but some flattened lesions still persisted. Two months after diagnosis, a few erosive lesions were still visible, mainly on the tongue’s edge (Figure 4). Histopathological examination of incisional biopsy of the lesions showed a mild inflammatory infiltrate, comprising plasma cells, macrophages, a few eosinophils and neutrophils, and no detectable leishmanial forms (Figure 5). Histopathology discarded neoplasia, *calcinosis circumscripta*, solar glossitis, vasculitis, amyloidosis, eosinophilic granulomas and burns. A real-time PCR assay for *Leishmania* DNA was carried out on lingual tissue and bone marrow, providing negative results in both cases. At the completion of treatment with allopurinol alone, i.e. seven months after diagnosis, the tongue appeared macroscopically normal (Figure 6) and IFAT was negative (titre < 1:20). Fifteen months after diagnosis this clinically favourable outcome continued (IFAT < 1:20).

CanL represents a severe veterinary medical problem, with dogs also playing an important epidemiologic role as a reservoir of infection to humans and further representing an experimental model of the disease [2,16]. Cases of oral leishmaniosis have been reported in humans with peculiar clinical presentations. These include a lingual fleshy tumour [17], granulomatous glossitis [18], a single lingual nodule associated to several palatal nodular lesions [19] and a voluminous lip tumour [20]. Furthermore, other related clinical conditions have included granulomatous plaques on the tongue [21], upper lip swelling and erythema, with the presence of crusts and scaling [22], painful ulcerations of the floor of the mouth or cheek mucosae [23] and fistulae and granulation of the hard palate [24].

Although it is known that canine cutaneous and visceral leishmaniosis manifestations are frequently diagnosed around the world [2], its oral presentation is quite rare. The first reference of tongue lesions in CanL was published by Font *et al.*, who described a *Leishmania*-infected dog with proliferative lesions on the tongue and oral cavity mucosa [5]. Saari *et al.* reported a 7-year-old male mongrel dog with a nodule protruding on the ventral surface of the tongue [6]. Amastigotes were observed after histological analysis of the excised mass. Blavier *et al.* described a 3-year-old Giant Poodle dog with several partially ulcerated lingual nodules, hyperkeratosis and non-healing ulcers on the footpads, with amastigotes detected on fine needle aspiration smears of those nodules [4]. Lamothe and Poujade reported a case of ulcerative glossitis in a 10-year-old mongrel dog, also presenting enlargement of the peripheral lymph nodes and splenomegaly. Amastigote forms were observed in biopsy samples obtained from this animal [9]. Manzillo *et al.* described an atypical situation of multiple red papules on the tongue surface in a 4-year-old female Doberman, which presented weight loss and multiple cutaneous, ocular and haematological alterations. Definitive diagnosis was obtained by IFAT and cytological identification of parasites in lingual lesions and bone marrow aspirates [25]. Parpaglia *et al.* showed a peculiar case of multiple non-ulcerated, dome-shaped nodular lesions on the tongue, as well as ocular and cutaneous lesions, enlargement of lymph nodes and mild splenomegaly in a 5-year-old intact female mongrel dog. Amastigotes were observed in histological samples of this animal [7]. A new report by Manzillo *et al*. described a multiple red, nodular lesions on the dorsal and lateral surfaces of the tongue. Diagnosis was made on the basis of a positive IFAT and amastigote observation in lingual lesions as well as in bone marrow [8].

Dogs reported by Saari *et al.*[6] and Lamothe and Poujade [9] had to be euthanized because of an adverse response to antimonial therapy and necrotic dermatitis, and poor physical conditions, respectively. The first dog described by Manzillo *et al.* died six months after diagnosis due to systemic disease [25]. The second case of Manzillo *et al*. had full remission of clinical signs at the end of a combined treatment with miltefosine and allopurinol [8].

It can be hypothesized that the dog described in the present report suffered a relapsing form of CanL, as suggested in similar cases by other authors [9]. However, parasites could have directly invaded the tongue mucosa through the bites or crushing of infected phlebotomine sandfly vectors, rather than having diffused from the skin or visceral organs [25,26]. Leucopenia and neutropenia might be explained by an increased tissue demand of white blood cells associated with the severe oral inflammation. Taking into account these clinico-pathological alterations and a possible relapse, the hypothesis of a false negative result for the IFAT titre (1:40) at the time of diagnosis should be considered. The dog presented a good response to treatment, with a complete regression of the tongue lesions. Nevertheless, a future relapse cannot be excluded and regular clinical re-evaluation should be maintained.

In conclusion, the reported case represents an uncommon clinical presentation of CanL, which should be included in the list of differential diagnosis for nodular and ulcerative glossitis in dogs.

## Competing interests

The authors declare that they have no competing interests.

## Authors’ contributions

CV, JR and CA collected samples, analysed data and drafted the manuscript; TS and JM managed the clinical case; ID helped to collect samples and revised the manuscript; MAP performed cyto- and histopathological analyses and revised the manuscript; LeC carried out PCR assays and revised the manuscript; LuC coordinated the study, analysed data and drafted the manuscript. All authors read and approved the final manuscript.
